# Saving Old Bones: a non-destructive method for bone collagen prescreening

**DOI:** 10.1038/s41598-019-50443-2

**Published:** 2019-09-26

**Authors:** Matt Sponheimer, Christina M. Ryder, Helen Fewlass, Erin K. Smith, William J. Pestle, Sahra Talamo

**Affiliations:** 1 Department of Anthropology, University of Colorado Boulder, Boulder, CO 80309 USA; 2 Department of Human Evolution, Max Planck Institute for Evolutionary Anthropology, 04103 Leipzig, Germany; 3 Department of Anthropology, University of Miami, Coral Gables, FL 33124-2005 USA; 4 Department of Chemistry “G. Ciamician”, University of Bologna, Via Selmi, 2, I-40126 Bologna Italy

**Keywords:** Archaeology, Palaeontology, Stable isotope analysis

## Abstract

Bone collagen is an important material for radiocarbon, paleodietary, and paleoproteomic analyses, but it degrades over time, making such analyses more difficult with older material. Collagen preservation between and within archaeological sites is also variable, so that much time, effort, and money can go into the preparation and initial analysis of samples that will not yield meaningful results. To avoid this, various methods are employed to prescreen bone for collagen preservation (e.g., %N, microporosity, and FTIR spectroscopic analyses), but these are often destructive and/or require exportation for analysis. Here, we explore near-infrared spectroscopy as a tool for gauging the collagen content of ground and whole bone from about 500 to 45,000 years ago. We show that a portable spectrometer’s ability to quantify collagen content and classify specimens by preservation status is comparable to that of other popular prescreening methods. Moreover, near-infrared spectroscopy is non-destructive and spectra can be acquired in a few seconds.

## Introduction

The persistence of organic molecules in bone has proven crucial for understanding the human past. Bone collagen (a common protein in bone and skin) from humans and our close kin has been used to radiocarbon (^14^C) date crucial events in human history, such as the peopling of the Americas^1^ and southeastern Europe^2^ and the disappearance of groups including the Neanderthals^3,4^. In fact, much of our understanding of the sequence of human history prior to the advent of writing and calendrical systems comes from radiocarbon dating of bone collagen or other organic materials from archaeological sites. Bone collagen is also a preferred material for stable isotopic paleodietary studies and has been used to document the emergence of maize agriculture^5^, the broadening of European *Homo sapiens’* resource base in the Upper Paleolithic^6^, and the importance of animal proteins in the diets of Neanderthals among other things^7^. It is also of increasing interest for paleoproteomic analyses, as it can be used to identify modern and ancient species and their phylogenetic histories even where ancient DNA studies are impossible or impractical^8–10^. Thus, it is fair to say that collagen is a material of signal importance for revealing the often murky human past, and that our ability to discern distant human behavior and evolutionary history will be roughly proportional to its preservation in the archaeological record.

Lamentably, collagen deteriorates over time, making it progressively more difficult to conduct analyses of these kinds as material gets older, although the speed of its degradation is heavily dependent on environmental conditions^11–14^. Moreover, preservation between and within individual archaeological or paleontological sites is highly variable. As a result, even recent sites may preserve little or no collagen, and ancient sites where collagen preservation is generally poor may have specimens that are surprisingly well preserved^11,12,15–17^. As a result, radiocarbon, paleodietary, and other archaeometric labs may need to destructively sample large numbers of specimens with the hope of finding a few suitable for analysis. This is not only ethically problematic, but it means that much time, effort, and money must go into the initial analysis and preparation of samples that will not yield meaningful results.

Consequently, there is intense interest in the development of methods to prescreen bone for collagen content while minimizing damage to specimens. Arguably, at least in the radiocarbon community, the standard method for determining a bone’s suitability for subsequent analysis is to take small subsamples (<5 mg) for elemental analysis where %N, and to a lesser extent C/N ratios, are used to estimate collagen preservation^16,18,19^. In general, samples with more than 0.76% N by weight are considered likely to retain more than 1% collagen, which is typically sufficient for ^14^C analysis^12,16,18^. Other prescreening methods for collagen preservation include mid-infrared or Raman spectroscopy which reveal information about a substance’s functional groups due to its interaction with electromagnetic radiation^17,20–29^. Although both elemental and spectroscopic techniques are clearly useful, they are often time-consuming, destructive, and/or typically require removal of bones from sites or museums to labs for analysis. Similar drawbacks exist for other potential prescreening techniques^25,30–37^.

There have been, however, a few attempts to circumvent these limitations. Most notably, portable Raman spectrometers with 1064 nm lasers were used to show that the ratio of peaks at 1450 cm^−1^ to 960 cm^−1^ is associated with collagen content and quality^17,38^. Additionally, qualitative non-destructive near-infrared (NIR) spectroscopy was used to classify 16 Holocene bones and four validation samples into poor and good collagen preservation groups^39^. Here, we build on the latter study to show that a portable and field ruggedized NIR spectrometer can be used not only to classify bones into groups by preservation status, but also to quantify percent collagen preservation (hereafter %coll) in ground and whole bone of Holocene to Late Pleistocene age. NIR spectroscopy has great potential for bone prescreening in that it is non-destructive, has a very fast speed of analysis (typically seconds), is readily miniaturized so that field-deployable instruments are widely available, and has a greater effective penetration depth than its spectroscopic siblings (millimeters as opposed to microns; Supplementary Fig. S1). Its ability to provide a deeper glimpse is especially important given that bone surfaces are often heavily modified post-depositionally^20,27,29,40,41^.

## Results & Discussion

**Figure 1:**
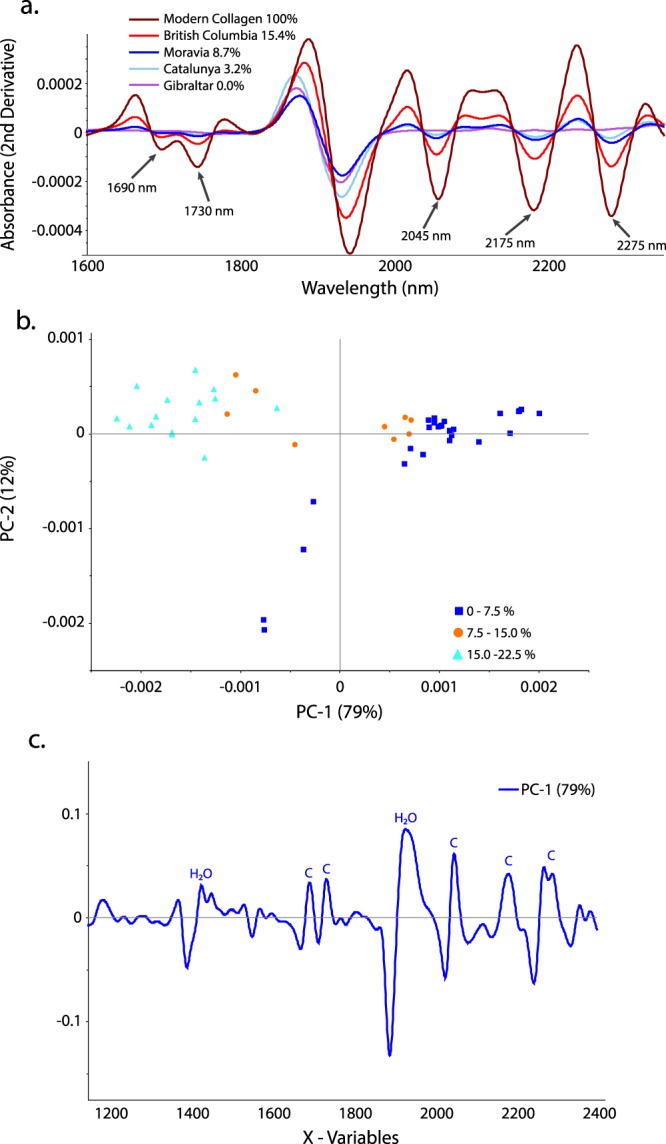
NIR bands reflect collagen content. (**a**) NIR absorbance spectra (second derivative; 51 points smoothing) of pure modern collagen (100% collagen; brown) and archaeological specimens from Gibraltar (0.0% collagen; pink), Catalunya (3.2% collagen; turquoise), Moravia (8.7% collagen; blue), and British Columbia (15.4% collagen; red). Multiple bands/regions (labelled) show expected directional shifts in accordance with % collagen. (**b**) PCA scores plot (PC1 and PC2) of the NIR spectra (780 nm to 2500 nm; second derivative) of 50 ground bone samples from archaeological sites. High collagen specimens (15.0% to 22.5%; turquoise triangles) are distinct from low collagen specimens (0% to 7.5%; blue squares) while samples with middling collagen contents (7.5% to 15%; orange circles) fall between these two groups. (**c**) PCA loadings plot showing influential variables for PC1. Bands/regions associated with collagen (labeled C) load on PC1.

The near-infrared spectra of archaeological specimens with differing collagen contents are clearly distinct and multiple bands/regions, including the first overtone of the C-H stretch at 1690–1750 nm, N-H stretching combinations at 2045 nm, the N-H bend second overtone and C=O stretch combinations at 2175 nm, and C-H combinations at 2275–2300 nm show expected differences related to %coll (Fig. 1a; Supplementary Fig. S2; see Methods)^42–45^. An exploratory principal component analysis (PCA) of the NIR spectra from 50 ground bone specimens from archaeological sites of Holocene age from the Old and New Worlds (Supplementary Table S1) reveals strong spectral differences between specimens with differing collagen contents (Fig. 1b; or C/N ratios in Supplementary Fig. S3) and bands/regions associated with collagen (above) are highly influential in the loadings plot for PC1 (Fig. 1c).

**Figure 2:**
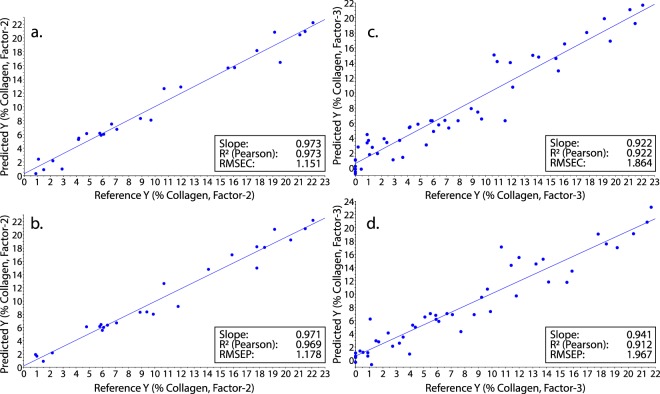
Predicting collagen preservation from NIR spectra. (**a**) Results of PLSR showing predicted versus actual %coll values for the 25 sample (ground bone) calibration set (R^2^ = 0.97). (**b**) Predicted versus actual %coll values using the calibration model on the 25 sample (ground bone) validation set (R^2^ = 0.97). (**c**) Results of PLSR showing predicted versus actual %coll values for the 49 specimen ground/whole bone calibration set (R^2^ = 0.92). (**d**) Predicted versus actual %coll values using the calibration model on the 48 sample validation set (R^2^ = 0.91).

Partial least squares regression (PLSR) on a calibration set (25 spectra) resulted in a two factor model that predicted %coll from spectral data remarkably well (R^2^ = 0.97, Root-Mean Square Error of Calibration [RMSEC] = 1.15) (Fig. 2a). The model also performed well when used to predict %coll from 25 independent validation specimens (R^2^ = 0.97; Root-Mean Square Error of Prediction [RMSEP] = 1.18) (Fig. 2b). Of the 21 specimens in the validation set with more than 3% collagen (the lower bound for quantitative FTIR in^24^), the model predicted more than 3% collagen 21 times (100% classification success). The model correctly predicted all four specimens with less than 3% collagen (100% classification success).

Given the success of the model with ground bone samples, we built a model that included ground and whole bone samples up to about 45 thousand years old (Supplementary Table S1). PLSR on spectra from a 49 sample calibration set generated a model that performed well (R^2^ = 0.92; RMSEC = 1.86; 3 factors; Fig. 2c) and predicted %coll in a 48 sample validation set equally well (R^2^ = 0.91; RMSEP = 1.97; Fig. 2d). Of the 32 specimens in the validation set with more than 3% collagen, the model predicted 30 correctly (94% classification success). Of the 16 specimens with less than 3% collagen it predicted 88% correctly. Most crucially, of the 32 specimens in the validation set that the model predicted had more than 3% collagen, every one had more than the 1% collagen typically required for radiocarbon and paleodietary analyses.

**Figure 3:**
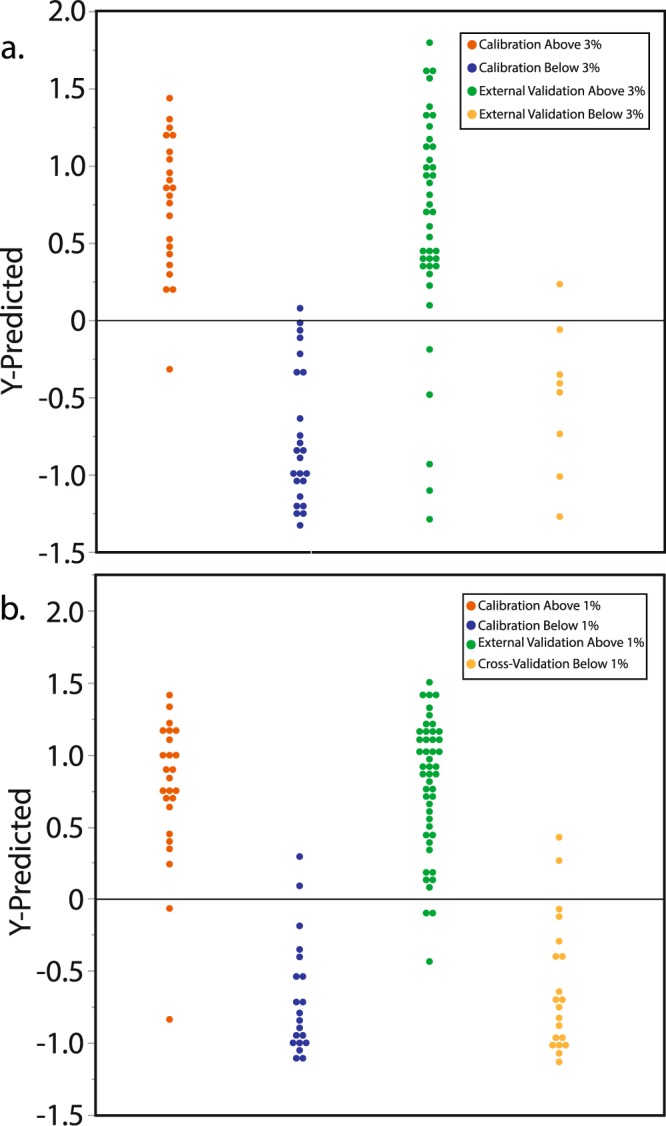
Predicting collagen preservation groups from NIR spectra. PLS-DA prediction scores showing predicted membership of specimens to the Sample (scores above 0) or Do Not Sample (scores below 0) groups. Specimens are grouped by their actual (not predicted) collagen yields. (**a**) PLS-DA prediction scores when the Sample group was defined as >3% collagen. Classification success for specimens in the validation set was 83%. (**b**) PLS-DA prediction scores when the Sample group was defined as >1% collagen. Classification success for specimens not included in the calibration dataset was over 90%.

This performance compares favorably with previous efforts to quantify %coll with portable Raman instruments^17,38^, especially when considering the greater temporal and spatial range of specimens employed here. In fact, the RMSEP here (1.18% and 1.92% for ground and ground/whole bone models respectively) is similar to the typical 1.6% intra-lab standard deviation for collagen extraction of the same specimen^46^. But while %coll estimates might prove crucial for some applications, such as when specimens with exceptional preservation are required, in most cases researchers are likely to ask the binary question, “Should I sample this specimen?” We used Partial Least Squares-Discriminant Analysis [PLS-DA] to test the utility of NIR spectroscopy for answering this question by classifying specimens into Sample versus Do Not Sample groups. When the Sample group was defined with calibration specimens having more than 3% collagen, classification success (i.e. external validation specimens with more than 3% collagen were assigned to the Sample group) was 83% (Fig. 3a). When the Sample group was defined by calibration specimens with more than 1% collagen (after^12,18,19^), classification success for validation samples was over 90% (Fig. 3b).

These results demonstrate that NIR spectroscopy can be used to ascertain collagen preservation status in archaeological bone from dozens of sites across the world which range in age from recent to more than 45,000 years old. It is likely, therefore, that this tool will prove generalizable. Many effective prescreening tools exist. Percent nitrogen can identify bones with more or less than 1% collagen better than 70% of the time^16,18,19^, and FTIR and Raman spectroscopy can achieve similar success sorting bones into good and poor preservation groups^17,20,22,24,26,28,38^. NIR spectroscopy’s niche will likely be applications where non-destructive analysis, high sample throughput, and/or penetration beyond surface contamination/alteration are paramount, as none of the existing methods meet all of these criteria.

For instance, sites often contain thousands of bones or fragments that might prove useful for radiocarbon, paleodietary, or paleoproteomic analyses. Even sites with relatively poor bone preservation (typically less than 1% collagen by weight) can contain specimens with reasonably intact collagen. For instance, of 50 bones analyzed from the Neanderthal site Zafarraya, three retained more than 4% collagen^16^. Acquisition of NIR spectra takes roughly five seconds per sample, so one could scan hundreds of samples to identify these rarities in a single afternoon.

Another benefit of this technique would be the ability to more adequately pick specific spots of individual bones for analysis. Archaeological bone is far from homogenous when it comes to collagen preservation^16,20,27,41^, yet typically only one small area is sampled to determine a bone’s suitability. It is easy to envision taking NIR scans of multiple spots on a bone (or taking hyperspectral images as in^39,47^) to pinpoint areas where sampling might be most fruitful. This is likely to be of greatest importance at sites where collagen preservation is especially poor, making the identification of even a few fragments with moderate preservation crucial. At a different scale, the speed and cost effectiveness of the technique could make it easier to address questions about inter- or intra-site variation in preservation and post-depositional processes^21,22,48–50^.

We are not suggesting that NIR spectroscopy should supplant %N or other spectroscopic techniques for addressing the question “Does this individual specimen have sufficient collagen for analysis?” However, the results presented here suggest that this tool has significant advantages over commonly employed techniques for answering the question, “Which of the hundreds or thousands of bones at this site (or in this collection) are especially well-preserved?” Near-infrared spectroscopy can do this form of cherry-picking quickly and inexpensively on site, and as a bonus, it should reveal environmental or conservation contaminants that must be removed for radiocarbon or isotopic paleodietary analyses^47,51–53^. This might save weeks of lab work, not to mention considerable analytical and labor costs. Most importantly, however, with such prescreening fewer specimens would be exposed to destructive analysis.

## Methods

### NIR spectroscopy

We used 50 archaeological ground bone samples of Holocene age to create our proof of concept NIR model. All samples were scanned (50 scans per sample) while still in their glass storage vials using a fiber-optic reflectance probe attached to a LabSpec 4 NIR spectrometer with a spectral range of 350 nm to 2500 nm. Subsequent data transformations and analyses were undertaken using Unscrambler X by CAMO Analytics. A Savitzky-Golay transformation (Derivative Order, 2; Polynomial Order, 3; Smoothing Points, 31) was performed to correct for additive and multiplicative effects in the spectral data^54^. Principal component analysis (PCA) was carried out to ascertain whether or not patterns relating to collagen preservation existed in the spectral data. After it was apparent that there were clear spectral differences relating to collagen content, the data were sorted by collagen yield and the even and odd samples were assigned to the calibration and validation sets respectively (25 calibration, 25 validation). Partial least squares regression (PLSR) was then used on the calibration set to create a model predicting %coll^55^. The model was then applied to the validation set to predict %coll and assign specimens to the above or below 3% groups. After this, the same procedures were used on samples of whole bone of Holocene and Late Pleistocene age which were scanned at exposed cross-sections (recent or natural breaks; Supplementary Fig. S4; ongoing work is assessing the method on all bone surfaces). These scans were then coupled with scans of ground bone (49 calibration, 48 validation) in the hope of producing a PLSR model that was less sensitive to the differing geometries and particle sizes of the ground and whole bone samples^54,55^. Bands associated with water (e.g., the O-H bend second overtone at 1940 nm) were excluded from all analyses. Both models shown here use the 1695–1750 nm and 2000–2300 nm spectral ranges because previous research has shown that they contain bands associated with proteins including collagen^42–45,56^, and because a PCA loadings plot of the spectra used here confirms that these bands are associated with collagen in our samples (Fig. 1c). It is worth noting, however, that it is possible to generate models with similar (or even greater) predictive power using three or fewer bands. Partial Least Squares-Discriminant Analysis (PLS-DA) was used to classify the ground and whole specimens into Sample and Do Not Sample groups defined as both above/below 3% collagen and above/below 1% collagen. Specimens were sorted by collagen yield to facilitate separation of calibration and validation sets. For the above/below 3% model, every fourth specimen below 3% collagen was left out for external validation as were two out of every three specimens with more than 3% collagen. This ensured similar sizes for the two groups in the model. For the above/below 1% model, no specimens below 1% collagen were left out for external validation as there were only 20 in total. For specimens above 1% collagen, one out of every three specimens (except the five with the highest % collagen) was included in the calibration set to keep the above and below 1% groups similar in size. The remaining samples were assigned to the external validation set. Cross-validation was also performed in Unscrambler X using the method random with 20 segments.

### Collagen extraction

Extractions of collagen for ground bone specimens of Holocene age took place in the Archaeological Stable Isotope Lab at the University of Miami following a modified version of Longin^57^. Weighed 0.5 g aliquots of coarsely ground (0.5–1.0 mm) cortical bone were placed in 50 ml centrifuge tubes, to which 30 ml of 0.2 M HCl was added. Tubes were placed in a rotator for 24 h, at which time the degree of demineralization was assessed. Samples requiring another 24 h to demineralize had their acid refreshed at this time. After demineralization, samples were rinsed to neutral and treated with 30 ml of 0.0625 M NaOH for a period 20 h. Samples were then rinsed to neutral and gelatinized for 48 h at 90 °C in 10^−3^ M HCl. The resulting gelatin was then filtered using 40 μm sterile single-use Millipore Steriflip ® vacuum filters, allowed to condense at 85 °C, frozen, and then freeze-dried. Collagen yields were then determined to assess the state of sample preservation.

Extractions of collagen for whole bone specimens of Pleistocene and Holocene age took place at the Max Planck Institute for Evolutionary Anthropology in Leipzig using a modification of Method C from^58^. About 0.5 grams of whole bone was decalcificied in 0.5 M HCl at 5 °C. Acid was refreshed up to twice per week until demineralization was complete. After demineralization, samples were rinsed with ultra-pure water to neutral pH and treated with 0.1 M NaOH at room temperature for 30 minutes to remove humic acids. This was followed by a 0.5 M HCl step to remove potential contamination from modern CO_2_ taken up by the NaOH. Samples were rinsed to neutral pH again with ultra-pure water and gelatinized for 20 h at 75 °C in 10^−3^ M HCl. The resulting gelatin was then filtered using precleaned Ezee filters (Elkay Labs UK) to remove larger particles and then ultrafiltered (precleaned Sartorius Vivapsin Turbo 15) to separate large (>30 kD) and small molecular weight fractions. The >30kD fraction was then freeze-dried for 48 hours after which collagen yields were calculated. The two methods of collagen extraction did not appreciably influence %coll predictions (Supplementary Fig. S5).

## Supplementary information

Saving Old Bones Supplementary Information

## Data Availability

Data for the analyses described herein are available in the Supplementary Information and on Figshare (https://figshare.com/s/7d1150732ab124e72a0d).
