# Supplementary material for: Saving Old Bones: a non-destructive method for bone collagen prescreening
Source: Sci Rep. 2019 Sep 26;9:13928. doi: 10.1038/s41598-019-50443-2 (PMC6763469; doi:10.1038/s41598-019-50443-2)
Supplement: Supplementary file 1 — Saving Old Bones Supplementary Information [file 41598_2019_50443_MOESM1_ESM.pdf]

Supplementary Information for:

Saving Old Bones: a non-destructive method for bone collagen prescreening

Matt Sponheimer[a*], Christina M. Ryder[a], Helen Fewlass[b], Erin K. Smith[a], William J. Pestle[c] & Sahra Talamo[b]

[a]*Department of Anthropology, University of Colorado Boulder, Boulder, CO 80309 USA*

[b]*Department of Human Evolution, Max Planck Institute for Evolutionary Anthropology, 04103 Leipzig, Germany*

[c]*Department of Anthropology, University of Miami, Coral Gables, FL 33124-2005, USA*

*Corresponding author

Email: matt.sponheimer@gmail.com

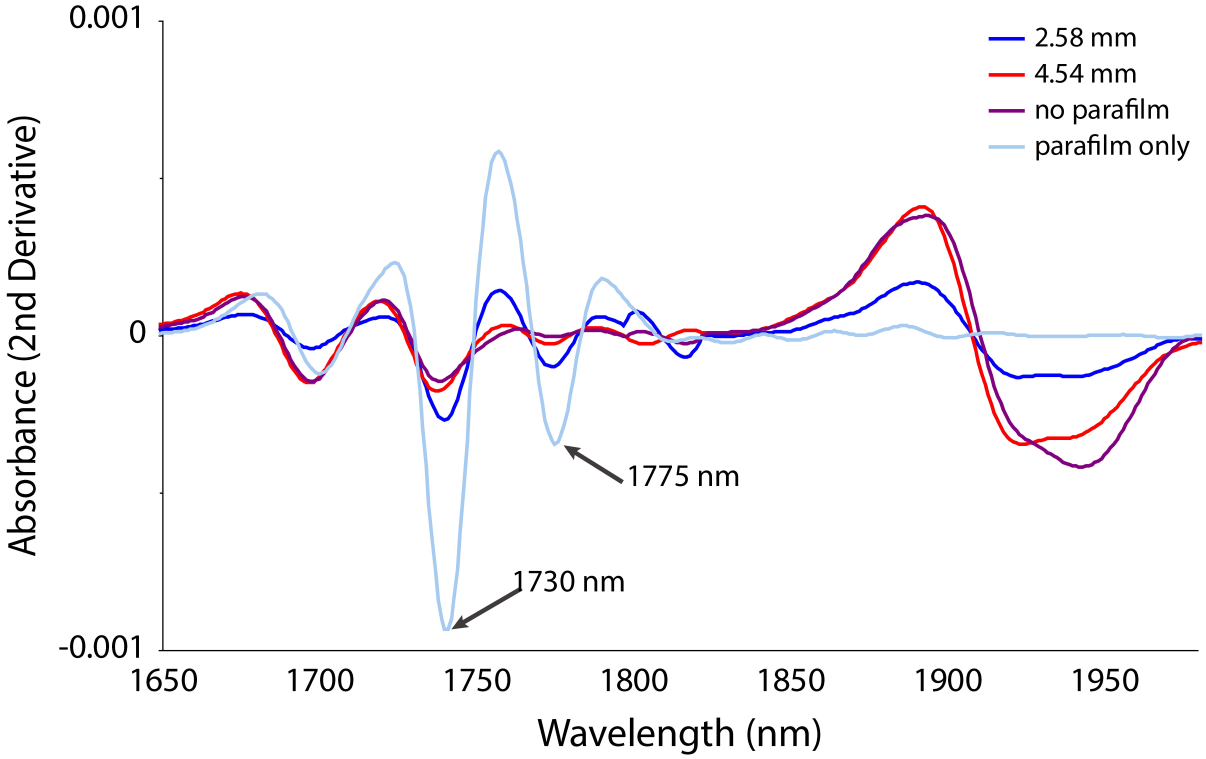

Supplementary Fig. S1: **NIR penetrance in bone.** NIR spectra (second derivative; 25 points smoothing) of parafilm (light blue), 2.58 mm of bone with parafilm beneath it (dark blue), 4.54 mm of bone with parafilm beneath it (red), and 4.54 mm bone with no parafilm beneath it (purple). All bone is from the same specimen. Note the strongly similar spectra for the 4.54 mm bone slice and the bone not underlain by parafilm. This suggests that the NIR signal is not readily extending beyond 4.54 mm in this spectral region. In contrast, parafilm peaks at 1730 nm and 1775 nm (among others) clearly influence the spectrum of the 2.58 mm bone slice. Thus, a penetration depth of 3-4 mm for this spectral region seems reasonable and is consistent with what has been found in other mineralized tissues[1].

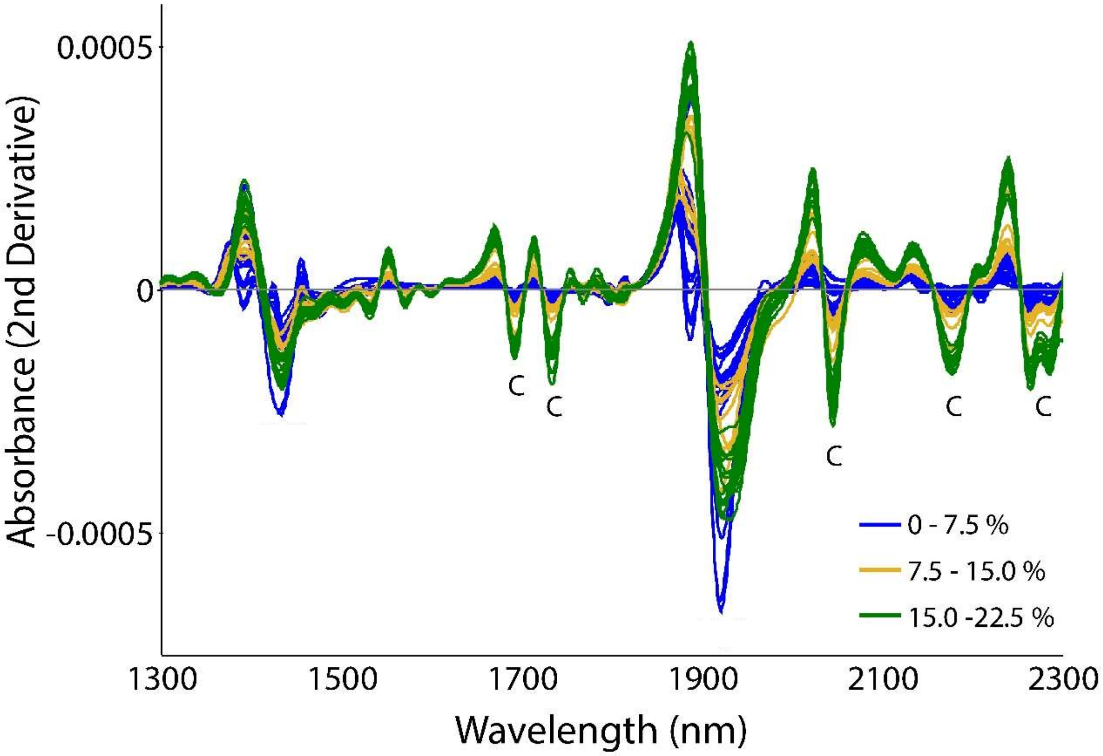

Supplementary Fig. S2: NIR spectra from ground bone samples reveal collagen content. NIR spectra (second derivative; 31 points smoothing) from all 50 ground bone specimens in this study. Green spectra are high %coll specimens (15.0-22.5%), gold spectra have less collagen (7.5-15%), while blue spectra have the lowest %coll (0.0-7.5%). Bands/regions that show strong patterning with %coll are labeled C.

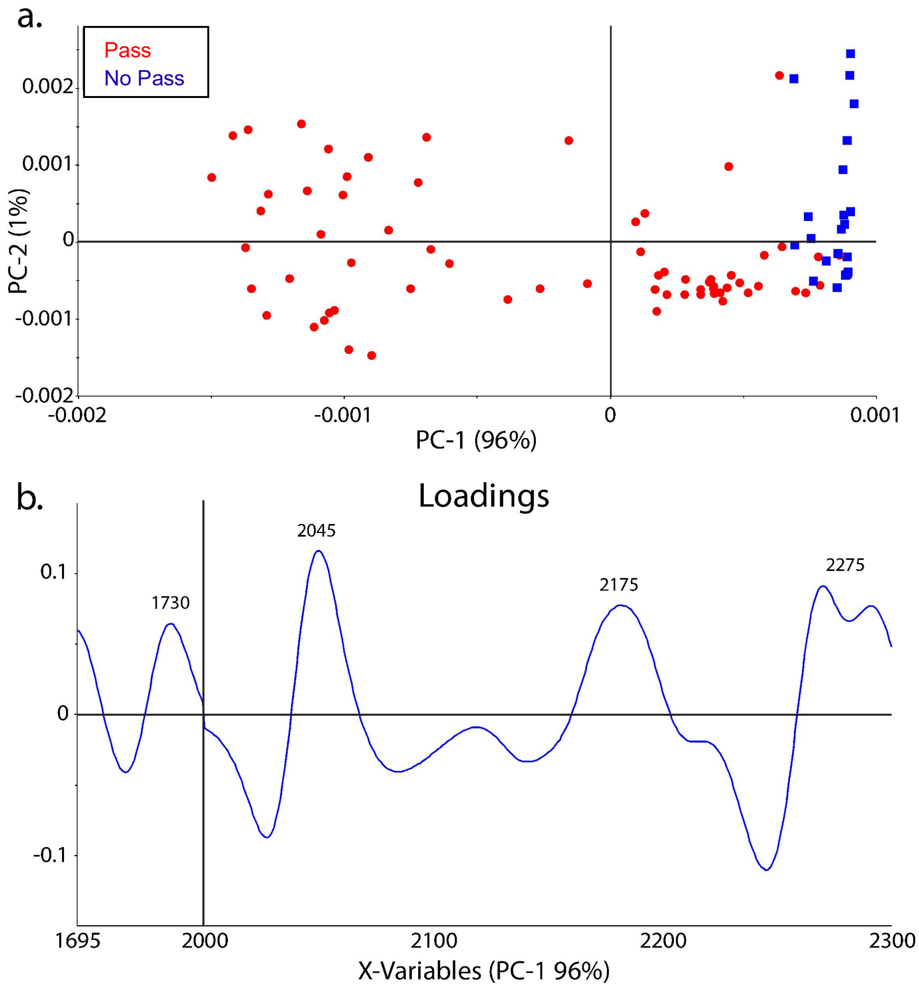

Supplementary Fig. S3: Predicting C/N ratio suitability from NIR spectra. A) PCA scores plot (PC1 and PC2) of the NIR spectra of archaeological bone samples. Specimens with C/N ratios between 2.9 and 3.6 are red circles (pass) and those that fall outside that range (or for which there was no preserved collagen to analyze) are blue squares[2,3]. B) PCA loadings plot showing that bands/regions associated with collagen load on PC1.

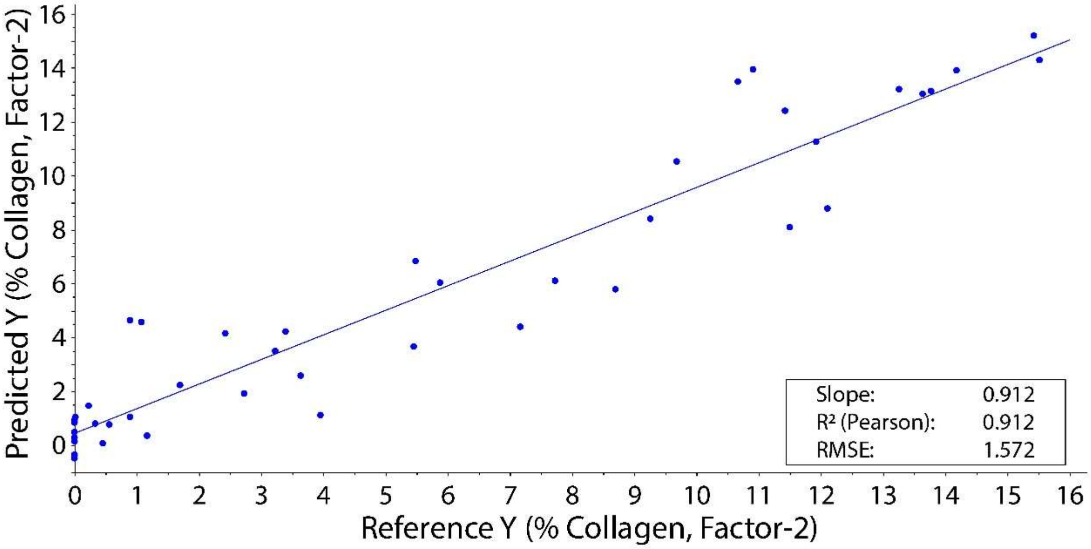

Supplementary Fig. S4: Predicting collagen preservation from NIR spectra of bone cross-sections. Results of PLSR showing predicted versus actual %coll values for 47 whole bone samples ranging in age from 2,000 to about 45,000 years old. Bones were scanned along previously exposed (naturally and otherwise) cross-sections. Studies focused on the external surface of bone are ongoing. The model's performance is good (R^2 = 0.91; RMSEC = 1.6; leave-one-out cross-validation R^2 = 0.90; Root Mean Square Error of Cross-Validation (RMSECV) = 1.7), although not as strong as the ground bone model (Figure 1a). This was expected. Collagen yield had been determined for both the ground and whole bone specimens (for radiocarbon and isotopic paleodietary studies) before spectra were acquired. Given the relative homogeneity of ground bone, the scans taken were almost certainly representative of the subsamples on which collagen determinations were made. Whole bone could not be scanned at the locations where %coll was determined as the sample is transformed during that process. Given that collagen preservation is heterogeneous within whole bone[4,5], it is likely that, at least in some cases, areas were scanned that had different %coll than the locations where collagen determinations were made. Thus, the ground bone and ground/whole bone models were thought to be better proofs of concept.

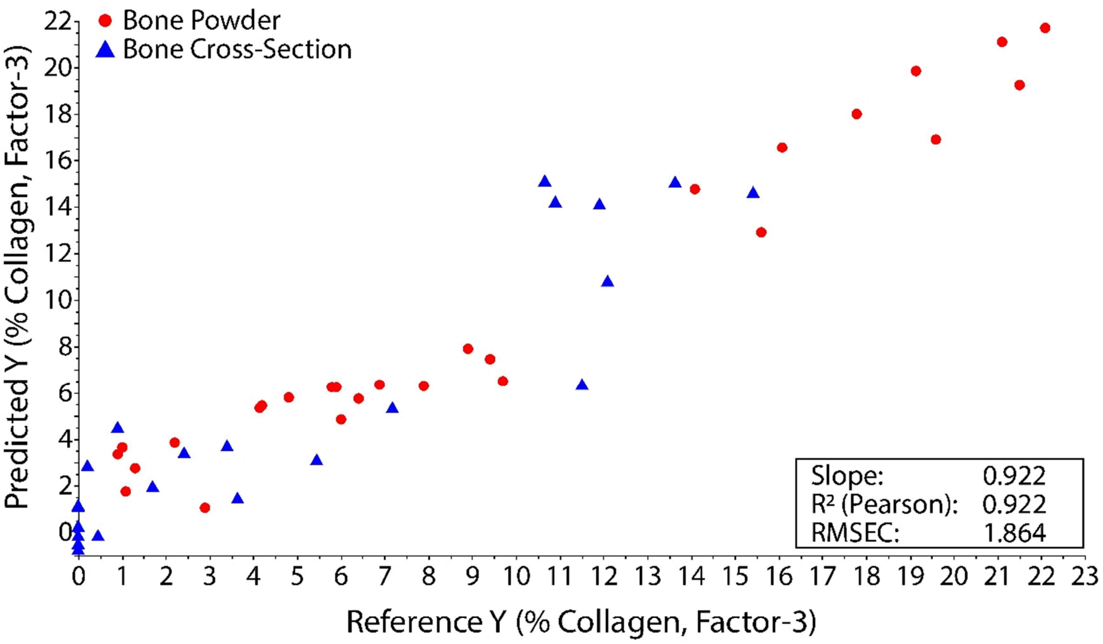

Supplementary Fig. S5: Influence of extraction method on %coll predictions. Results of PLSR showing predicted versus actual %coll values for ground and whole bone samples ranging in age from 2,000 to about 45,000 years old. There are no clear differences between specimens that were ultrafiltered (blue triangles) and those that were not (red circles), which is perhaps surprising given that ultrafiltration removes lower molecular weight material (<30 kDa fractions) resulting in lower collagen yields[6–8]. In this instance, however, the ultrafiltered material was extracted from bone chunks, which tend to produce higher yields than ground bone[7,9], and the non-ultrafiltered bone was extracted from powder. We suspect that the lack of an obvious difference above is a consequence of the ultrafiltered/non-ultrafiltered and ground/chunk effects partially offsetting each other.

Supplementary Table S1. Specimen IDs, areas of origin, approximate ages, and collagen yields (%wt) for specimens included in this study. Italicized specimens were analyzed as ground bone.

**Specimen**	**Country/Territory**	**Thousands of Years BP (approx)**	**Collagen Yield (wt%)**
R1(1)	Italy	45	0
R2(1)	Italy	45	0.2
R3(1)	Italy	45	0.5
R4	Italy	45	0
R5	Italy	45	0.3
R6	Italy	45	1.1
R7	Italy	45	0
R8	Italy	45	0.6
R9	Mongolia	45	11.9
R10	Serbia	25-45	7.2
R11	Serbia	25-45	13.8
R12	Serbia	25-45	4
R13	Serbia	25-45	9.3
R14	Serbia	25-45	9.7
R28	Gibraltar	45	0.9
R29	Italy	25	0.9
R30	Spain	50-30	5.5
R31	Spain	50-30	5.9
R32	Spain	50-30	2.7
R33	Spain	50-30	1.7
R34	Spain	50-30	3.2
R35	Spain	50-30	1.2
R36	Spain	50-30	0
R37	Italy	50-40	0
R38	Italy	50-40	0
R39	Italy	50-40	0
R40	Italy	50-40	0
R41	Italy	50-40	0
R42	Hungary	14-13	7.7
R43	Hungary	14-13	3.6
R44	Hungary	14-13	5.5

R45	Hungary	14-13	2.4
R46	Gibraltar	45	0
R47	Gibraltar	45	0
R48	Gibraltar	45	0
R49	Canada	2	13.3
R50	Canada	2	10.9
R51	Canada	2	15.4
R52	Canada	2	12.1
R53	Canada	2	15.5
R54	Canada	2	14.2
R55	Canada	2	13.6
R56	Mongolia	33	11.4
R57	Mongolia	33	10.7
R1	Austria	>50	11.5
R2	Czech Republic	26	8.7
R3	Czech Republic	26	3.4
A-110	*Puerto Rico*	*1*	*9.4*
A-13	*Puerto Rico*	*1*	*4.2*
A-18	*Puerto Rico*	*1*	*4.4*
A-31	*Chile*	*2*	*0.9*
A-45	*Puerto Rico*	*1*	*6.1*
A-47	*Puerto Rico*	*1*	*5.9*
A-52	*Puerto Rico*	*1*	*6.7*
A-53	*Puerto Rico*	*1.5*	*6.4*
A-56	*Puerto Rico*	*1.5*	*6*
A-58	*Chile*	*1.5*	*1.3*
A-60	*Puerto Rico*	*1.5*	*2.4*
A-63	*Puerto Rico*	*1*	*9.7*
A-83	*Puerto Rico*	*1*	*7.9*
C-18	*Puerto Rico*	*1.5*	*9.9*
F-18	*Puerto Rico*	*1*	*1*
F-22	*Puerto Rico*	*1*	*5.1*
F-28	*Puerto Rico*	*1*	*5.9*
F-3	*Puerto Rico*	*1*	*4.2*
F-32	*Puerto Rico*	*1*	*2.2*
F-35	*Puerto Rico*	*1*	*7.1*
F-42	*Puerto Rico*	*1*	*6.9*
F-5	*Puerto Rico*	*1*	*4.2*
F-55	*Puerto Rico*	*1*	*5.8*

F-63	*Iraq*	*4*	*1.1*
F-71	*Chile*	*2.5*	*17.8*
F-76	*Chile*	*2*	*21.7*
F-92	*Chile*	*1*	*11.8*
G-20	*Chile*	*1*	*12*
G-21	*Chile*	*1*	*16.1*
G-43	*Chile*	*1*	*0.9*
G-46	*Chile*	*1*	*19.2*
G-59	*Kenya*	*0.5*	*3.5*
G-8	*Chile*	*1*	*19.6*
H-101	*Chile*	*1*	*20.4*
H-104	*Chile*	*1*	*22.1*
H-105	*Chile*	*1*	*4.8*
H-112	*Chile*	*1*	*1.5*
H-13	*Chile*	*1*	*18.4*
H-46	*Chile*	*2*	*10.7*
H-78	*Chile*	*1*	*15.6*
H-80	*Chile*	*1*	*8.9*
H-9	*Chile*	*1*	*21.4*
H-90	*Chile*	*0.5*	*21.5*
H-95	*Chile*	*0.5*	*19.2*
I-110	*Chile*	*2.5*	*21.1*
I-2	*Chile*	*1.5*	*2.9*
I-25	*Chile*	*1.5*	*15.9*
I-48	*Puerto Rico*	*1.5*	*1.7*
J-2	*Chile*	*2.5*	*14.1*
J-3	*Chile*	*2.5*	*17.8*

Supplementary References

1. McGoverin, C. M., Lewis, K., Yang, X., Bostrom, M. P. G. & Pleshko, N. The contribution of bone and cartilage to the near-infrared spectrum of osteochondral tissue. *Appl. Spectrosc.* **68**, 1168–1175 (2014).
2. DeNiro, M. J. Postmortem preservation and alteration of in vivo bone collagen isotope ratios in relation to palaeodietary reconstruction. *Nature* **317**, 806–809 (1985).
3. Ambrose, S. H. Preparation and characterization of bone and tooth collagen for isotopic analysis. *J. Archaeol. Sci.* **17**, 431–451 (1990).
4. Jacob, E. *et al.* Nitrogen content variation in archaeological bone and its implications for stable isotope analysis and radiocarbon dating. *J. Archaeol. Sci.* **93**, 68–73 (2018).
5. Lebon, M., Zazzo, A. & Reiche, I. Screening in situ bone and teeth preservation by ATR-FTIR mapping. *Palaeogeogr. Palaeoclimatol. Palaeoecol.* **416**, 110–119 (2014).
6. Sealy, J., Johnson, M., Richards, M. & Nehlich, O. Comparison of two methods of extracting bone collagen for stable carbon and nitrogen isotope analysis: comparing whole bone demineralization with gelatinization and ultrafiltration. *J. Archaeol. Sci.* **47**, 64–69 (2014).
7. Pestle, W. J., Crowley, B. E. & Weirauch, M. T. Quantifying inter-laboratory variability in stable isotope analysis of ancient skeletal remains. *PLoS One* **9**, e102844 (2014).
8. Brown, T. A., Nelson, D. E., Vogel, J. S. & Southon, J. R. Improved Collagen Extraction by Modified Longin Method. *Radiocarbon* **30**, 171–177 (1988).
9. Fewlass, H. *et al.* Pretreatment and gaseous radiocarbon dating of 40-100 mg archaeological bone. *Sci. Rep.* **9**, 5342 (2019).
